# Perceived social approval and condom use with casual partners among youth in urban Cameroon

**DOI:** 10.1186/1471-2458-11-632

**Published:** 2011-08-06

**Authors:** Ronan Van Rossem, Dominique Meekers

**Affiliations:** 1 Department of Sociology, Universiteit Gent, Korte Meer 3-5, 9000 Gent, Belgium; 2 Department of International Health and Development, Tulane University School of Public Health and Tropical Medicine, New Orleans, LA, USA

## Abstract

**Background:**

HIV prevention programs targeting youth often emphasize the role of peers, and assume that youths will model their behavior after their peers'. We challenge this view; we argue that adopting a given behavior requires social approval, and that youths do not necessarily turn to peers for such approval. This study analyzes survey data on youths in urban Cameroon to 1) identify which type of persons youths look to for social approval, and 2) establish how important social approval by these persons is for condom use among youths.

**Methods:**

We analyzed data from three survey waves (2000, 2002, and 2003) of a reproductive health survey conducted among urban Cameroonian youth (aged 15-24). Only respondents who reported having at least one casual partner in the past year were retained for the analysis. Bivariate analyses and structural equation modeling were used to examine relationships among perceived social approval, attitudes towards condoms and condom use.

**Results:**

The data show that only 3% of youths named their friends as people whose opinion they valued, while 93% mentioned family members. The perceived approval of condom use by these persons had a significant positive effect on the frequency of condom use among youths. The frequency of condom use was also affected by the respondents' attitudes toward condom use, the range of persons with whom they discussed reproductive health matters, whether they were enrolled in school, socioeconomic status, their self-efficacy, perceived severity of AIDS, risk perception and sexual risk behavior. The perceived social approval of condom use and the respondents' own condom attitudes were correlated.

**Conclusions:**

Our analysis demonstrates that perceived social approval facilitates the adoption of condom use among urban Cameroonian youth. However, youths tend to value the opinions of family members much more than the opinions of their peers. These results suggest that interventions targeting youths should not focus exclusively on peers but should also include other groups, such as parents and community leaders.

## Background

Peer education is considered an effective technique for HIV prevention programs to reach and influence youth [1]. The basic idea is that the adoption of safer reproductive health behaviors is affected by the perceived normative environment and the individual's willingness to conform to that environment [2]. It is often assumed that for youths this normative environment is primarily shaped by other youth. This paper examines the effects of perceived social approval on condom use with casual partners among Cameroonian youth, and argues that peers may be less effective in bringing forth behavior change than is often assumed. Behavior change requires social approval, and peers may simply not be the most relevant reference group for youths.

Many Cameroonian youths engage in risky sexual behaviors, such as having sex with casual partners or having unprotected intercourse, which may lead to unplanned pregnancies and STIs, including AIDS [3-12]. Despite the risks, consistent condom use has remained fairly low [3,7,13]. Among unmarried youth in Yaoundé, only 24% of men and 16% of women reported frequently using condoms [14]. Another study estimated condom use among youths at 16% for women and 31% for men [15]. The *Demographic and Health Surveys*, however, show that condom use increased between 1991 and 2004 [16]. While in 1991 only 1.0% of sexually active women (ages 15-49) reported to currently use condoms, this increased to 3.5% in 1998 and 9.7% in 2004. For women aged 20-24 the proportion currently using condoms increased from 1.0% in 1991 to 6.2% in 1998 and 16.0% in 2004.

The *100% Jeune *program, a social marketing program targeting youth aged 15-24 in Yaoundé and Douala, Cameroon's two major cities, was initiated and implemented by *Programme de Marketing Social au Cameroon *(PMSC) from 2000 onward [17,18]. It aims to motivate urban youths to adopt safer reproductive health behaviors, including abstinence and condom use. A wide range of social marketing techniques are used, including mass media campaigns, education sessions and other peer education techniques [19-22]. Initially the program focused exclusively on youths, but subsequent phases of the program also sought to stimulate parent-youth communication [17].

Several theories of behavior change emphasize the importance of social learning [23]. However, most theories focus on the cognitive dimensions of the social environment. That is, it is assumed that youths are influenced by peers who act as role models and opinion leaders and that these influences will help create attitudinal and behavior change. However, within the African context such emphasis on peers is by no means self-evident. The presumed importance of the role of peers is based on the presumption that traditional authority systems, such as the family and the community, are declining due to modernization processes [2,24-27]. It is assumed that as decisions about one's sexual behavior--such as whether to engage in intercourse or to use condoms-- become more individualized, peers become more important as opinion leaders. However, such arguments may underestimate the tenacity of traditional structures, which are integrated in the modernization process rather than being completely discarded. Hence, modernization does not necessarily imply the complete decline of traditional family and community structures [28]. Family and community structures can remain important frames of reference, even in the more modernized segments of these societies and therefore remain relevant contexts for decisions concerning reproductive health behavior. Furthermore, opinion leaders have a dual function; they not only disseminate information, but also express their approval/disapproval of various behaviors.

Social capital, social network and social control theories [29-31] conceptualize the social environment not only as a source of information, but also as a source of emotional support, practical support, identity, status, approval, and, perhaps even more importantly, social control. Behavior deemed inappropriate by others may be negatively sanctioned. These sanctions may vary from mild disapproval, over loss of social status and stigmatization, to social exclusion. Thus, reproductive health behavior needs cultural legitimation. Adopting safe sex practices may be difficult in the face of widespread social disapproval. For instance, Cameroonian women who carry condoms are stigmatized as sluts or prostitutes [32]. A supportive social environment, to the contrary, may facilitate the adoption of safe sex practices. Studies on Ghana, Zimbabwe, and Nigeria found a positive relationship between perceived social support and condom use [33-36]. A study among Cameroonian youths found that parental support increased the likelihood that youths had ever used condoms [17]. This suggests that family members and other adults may be more relevant than peers, when it comes to social approval of condom use.

Although peers may be important for the diffusion of new ideas, other actors, such as parents, other family members or religious leaders, may be more important for the acceptance of these ideas or the cultural legitimacy of such behavior [37]. In societies where the (extended) family is still an important principle of social organization and where social organization tends to be hierarchical, the opinion of family members or community leaders is likely to matter most. In many African societies, parents, family and community members still feature prominently in the everyday life of the adolescents. Because adolescents still depend on them financially, emotionally, socially as well as culturally, these actors continue to influence adolescent behavior through (anticipated) positive and negative sanctions. This study identifies actors whose opinions are valued by youths in urban Cameroon, and looks at the effect of their perceived social approval on condom attitudes and condom use with casual partners.

The literature has identified other factors that also affect condom use. For instance, self-efficacy is a central concept in both social learning theory and the theory of planned behavior [38,39], and is known to increase the likelihood of condom use [17,33,40-42].

Youths' attitudes toward condoms, the perceived effectiveness of condoms for family planning and STI prevention, and the perceived advantages and disadvantages of condom use are also important determinants of condom use. Youths who have positive attitudes toward condom use are more likely to use them, while those who have negative attitudes or perceptions are less likely to do so [17,43,44,44].

Risk perception is another factor affecting condom use. Increased awareness of the severity of the AIDS epidemic leads to increased condom use [17,45]. Youths who consider themselves at high risk for HIV infection are also more likely to adopt protective behaviors [17,33,34,42,46,47].

However, condom use is also dependent on the community context. For instance, condom availability (ease of access) is known to facilitate condom use [17,37,48-51]. The cultural acceptance of condom use and the community attitude and openness toward reproductive health issues also affect use. In communities where reproductive health issues can be discussed freely and where condom use is condoned or approved of, individuals are more likely to actually use them [37,52].

## Methods

### Samples

This study uses data from the 2000, 2002 and 2003 waves of the Cameroon Adolescent Reproductive Health Survey. These surveys were commissioned by the *Programme de Marketing Social au Cameroun *(*PMSC*), and implemented by the *Institut de Recherche et des Études de Comportements *(*IRESCO*) and the *Forum Camerounais de Psychologie *(*FOCAP*) [8,53,54], as part of the evaluation of the *100% Jeune *program. The data is not public. The surveys targeted youths aged 15 to 24 living in Cameroon's two major cities Yaoundé and Douala. A stratified sampling design was used in which neighborhoods were selected with probability of selection proportional to size (PPS), based on the number of inhabitants aged 15-24. Within each neighborhood, a similar PPS sampling procedure was used to select enumeration areas. At the next step, households were randomly selected in each enumeration area, and within each household one eligible person was randomly selected. In total 2,096, 3,536 and 3,627 respondents were interviewed in the three survey waves, respectively [for more detailed information, see reference [19]]. In each wave a separate sample was drawn. Informed consent was obtained from each of the participants and all necessary measures taken to guarantee the confidentiality of the information. Our analysis is restricted to respondents with a casual partner during the past 12 months, reducing the working samples to 378 (18%), 609 (17%) and 602 (17%), respectively.

### Variables

#### Condom use

The main outcome variable consists of the respondent's self-reported frequency of condom use with casual partners (never, sometimes, often, always) (see Table 1).

**Table 1:** Descriptive statistics for the three samples (Cameroon, 2000, 2002, & 2003)

	Survey				
			
	2000	2002	2003	Total	Test for
Frequency condom use with casual partners					χ^2^(6) = 109.61***
Never	18.0%	14.0%	6.0%	11.9%	
Sometimes	19.8%	22.3%	16.4%	19.5%	
Often	19.8%	12.6%	6.6%	12.1%	
Always	42.3%	51.1%	70.9%	56.5%	
Respondent's attitude towards condoms	0.71	0.71	0.77	0.73	E^2 ^= 5.9%***
	(0.13)	(0.14)	(0.13)	(0.13)	
MVPs					χ^2^(18) = 25.6
Father	26.6%	32.7%	31.7%	30.9%	
Mother	43.4%	38.4%	36.5%	38.9%	
*Parents*	*69.9%*	*71.1%*	*68.3%*	*69.8%*	
Grandfather	1.6%	2.0%	2.8%	2.2%	
Grandmother	1.1%	0.7%	0.8%	0.8%	
Family member	21.3%	21.0%	19.9%	20.7%	
*Other family*	*23.9%*	*23.6%*	*23.6%*	*23.7%*	
**Total kin**	**93.9%**	**94.7%**	**91.9%**	**93.4%**	
Teacher	0.5%	0.2%	0.5%	0.4%	
Religious leader	0.3%	0.5%	0.5%	0.4%	
Friend	2.9%	3.8%	3.3%	3.4%	
Star	0.3%	0.2%	0.0%	0.1%	
Other	2.1%	0.7%	3.8%	2.2%	
**Total non-kin**	**6.1%**	**5.3%**	**8.1%**	**6.6%**	
Perception of MVP's attitude towards condoms					χ^2^(8) = 18.60*
Disapproves completely	5.9%	3.6%	2.3%	3.7%	
Disapproves	10.6%	10.8%	9.0%	10.1%	
Neither pro or against	17.0%	12.6%	17.6%	15.6%	
Approves	33.8%	38.8%	33.9%	35.7%	
Approves completely	32.7%	34.2%	37.2%	35.0%	
*Social environment*					
Discussed either FP, STI or AIDS, past 12 months					χ^2^(2) = 16.72***
No	22.8%	26.4%	16.8%	21.9%	
Yes	77.2%	73.6%	83.2%	78.1%	
Range of persons respondent discussed FP, STD, AIDS with	0.06	-0.05	0.18	0.06	E^2 ^= 1.1%***
	(0.95)	(0.90)	(1.03)	(0.97)	
*Socio-demographic characteristics*					
Sex of respondent					χ^2^(2) = 0.03
Man	78.8%	79.0%	78.6%	78.8%	
Woman	21.2%	21.0%	21.4%	21.2%	
Age of respondent	19.97	19.93	20.31	20.08	E^2 ^= 0.6%*
	(2.33)	(2.36)	(2.41)	(2.38)	
Number of assets owned by HH	3.87	3.67	3.51	3.66	E^2 ^= 0.6%**
	(1.88)	(1.83)	(1.55)	(1.75)	
Respondent still in school					χ^2^(2) = 20.14***
Not in school	45.5%	56.0%	43.9%	48.9%	
In school	54.5%	44.0%	56.1%	51.1%	
*Self-efficacy*					
Can convince casual partner to use condom					χ^2^(2) = 13.48**
No	11.9%	12.3%	6.5%	10.0%	
Yes	88.1%	87.7%	93.5%	90.0%	
Can avoid HIV					χ^2^(2) = 20.73***
No	4.2%	7.2%	1.8%	4.5%	
Yes	95.8%	92.8%	98.2%	95.5%	
*Perceived severity*					
Aids societal problem					χ^2^(4) = 4.05
A serious problem	90.5%	88.3%	91.7%	90.1%	
A problem like another	8.2%	10.3%	7.3%	8.7%	
Not really a problem	1.3%	1.3%	1.0%	1.2%	
AIDS is curable/not terminal	1.38	1.17	1.17	1.22	E^2 ^= 1.2%***
	(0.74)	(0.79)	(0.80)	(0.79)	
*Perceived risk*					
Perceived AIDS risk					χ^2^(6) = 34.84***
No risk	5.8%	7.1%	10.3%	8.0%	
Low risk	10.3%	14.1%	18.4%	14.9%	
Moderate risk	9.0%	10.3%	13.8%	11.3%	
High risk	74.9%	68.5%	57.5%	65.8%	
*Sexual risk behavior*					
Number of partners, past 12 months	4.12	4.34	3.84	4.10	E^2 ^= 0.4%
	(3.62)	(3.95)	(3.44)	(3.69)	
Frequency of intercourse, past month	2.85	4.08	2.89	3.34	E^2 ^= 1.8%***
	(4.40)	(4.67)	(4.02)	(4.41)	
*Access to condoms*					
Time required to obtain condom	8.63	8.57	5.92	7.58	E^2 ^= 1.6%***
	(10.88)	(12.08)	(7.22)	(10.27)	

*N*	378	609	602	1589	

significance: *: *p *< 0.050; **: *p *< 0.010; ***: *p *< 0.001

#### Attitude toward condom use

The respondent's attitudes towards condoms and condom use was measured using an index created from 16 items (see Appendix 1) with which the respondent could agree (1) or disagree (0) or say he/she doesn't know (0.5). The index equals the mean item score, and a higher score indicates a more positive attitude toward condoms and condom use.

#### Perceived social approval

Respondents were asked to name someone whose opinion they valued a lot. This question was general and did not refer to any specific issue. Subsequently, respondents were asked how this "most valued person" (MVP) would react if he/she were to discover that the respondent used condoms. Possible answers ranged from disapprove completely (0) to approve completely (4). It is noted that the level of perceived social approval does not necessarily correspond with the actual level of approval. Indeed, some studies suggest that respondents tend to exaggerate homophily with their friends and relatives [55,56]. That is, respondents may incorrectly assume that the people they associate with have attitudes that are similar to their own. In other words, perceived social approval may overestimate actual levels of social approval.

#### Control variables

*Socio-demographic controls *included the respondent's sex and age (range 15-24), and whether they are still in school. A socioeconomic status (SES) asset index was created based on household possession of 9 items: bicycle, motorcycle, car, van, radio-tape player, radio, TV, tape recorder and fridge.

Two variables measured the *openness of the community toward reproductive health issues *A first variable captured whether the respondent discussed family planning, STI or AIDS prevention issues in the past year. The second variable counted the different types of persons these issues were discussed with: friend, spouse, parent, doctor, etc. This variable was standardized.

*Condom access *was operationalized as the estimated time needed to obtain a condom (in minutes; truncated at 60 minutes maximum).

We used two indicators of *self-efficacy*: whether respondents believed one can do something to avoid AIDS, and whether respondents believed they would be able to convince their partner to use condoms.

Two variables were used to measure the *perceived severity *of the AIDS epidemic. The first variable measures awareness that AIDS is a terminal disease: whether the respondent believed that 1) AIDS can be cured, and 2) that one can survive AIDS. The second variable measures respondents' opinion about the severity of AIDS in their community (a serious problem; a problem like another; not really a problem).

To measure youths' *perceived risk *of HIV infection, respondents were asked whether, if they were not to use condoms, they considered themselves to be at high risk, moderate risk, low risk or no risk of contracting AIDS.

Because high-risk sexual behavior is also a determinant of condom use [57], two indicators of sexual risk behavior were included: the respondent's number of partners (spouse, regular, and casual) during the past 12 months, and the number of sex acts during the past month.

### Statistical analysis

We test whether the perceived attitude of MVPs towards condom use affects the respondent's attitude towards condoms and his/her condom use. To control for the tendency to overestimate the homophily between one's own attitudes and those of MVPs we allow mutual effects between the attitudes of MVPs and respondents. To estimate this non-recursive model LISREL (version 8.72) was used [58]. To facilitate estimation, an instrumental variable for the perceived attitudes of MVPs towards condom use was added to the model. This variable equals the mean of the perceived social approval score for the type of person the respondent valued most. To assure the necessary independence of this variable, the respondent was not included in the calculation of this mean. For instance, if a respondent named his/her father as MVP, this variable consisted of the mean perceived social approval scores for all respondents in the wave that named their fathers as MVP, except for the respondent him/herself.

Since three independent surveys are included in the analysis, a multigroup analysis was deemed appropriate. In the initial model the regression coefficients for the three waves (2000, 2002, & 2003) were all constrained to be equal across all waves. In subsequent steps, these equality constraints were lifted for coefficients with large modification indices, while non-significant coefficients were fixed to zero. As the perceived attitude of the MVPs toward condom use and the frequency of condom use were both measured using ordinal variables, polychoric and polyserial correlations (and covariances) were used in the analysis for these variables. The final model fits the data well.

## Results

### Descriptive statistics

Because our working samples are restricted to those youths who reported having at least one casual partner in the year prior to the survey, all three samples consist of approximately 80% men and 20% women (see Table 1). The mean age of the respondents increased slightly from 20.0 years in 2000 and 2002 to 20.3 years in 2003. The assets index also indicates that on average, respondents became poorer between 2000 and 2003. The 2002 sample also contains significantly fewer respondents who are still in school than the 2000 and 2003 samples.

Table 1 shows that condom use increased over the course of the three surveys. Specifically, the percentage of youths who reported often or always using condoms increased sharply from 62% in 2000 to 78% in 2003. Likewise, the percentage of youth who had positive attitudes toward condoms increased significantly between 2000 and 2003, but this change is considerably less outspoken than the one for reported condom use. Perceived social approval of condom use also increased significantly between 2000 and 2003.

Both indicators of *self-efficacy*, whether one can do something to avoid AIDS, and whether respondents believe they can convince their partner to use condoms, show a significant increase from the 2000 to the 2003 survey, but both with a dip in the 2002 survey. However, over the same time we also observe a significant decline of the proportion of respondents who consider themselves to be at a high risk for AIDS, from 75% in 2000 to 58% in 2003.

The mean number of partners during the past year is 4.1, but 57% of respondents reported having three or fewer partners, while only 5% reported having 10 or more partners. The mean number of sex acts during the past month was 3.3. 28% of respondents abstained in the past month, while 8% reported having 10 or more sex acts.

### Identification of MVPs

The overwhelming majority of respondents mentioned that the people whose opinion they valued most were family members (93%, see Table 1). While 70% mentioned a parent as their MVP, less than 4% mentioned friends, i.e., peers. The finding that youths are more likely to value the opinions of their parents than those of their friends raises questions about programs that emphasize the role of peers.

Between 2000 and 2003, the percentage of youths who believed that their MVP had a positive attitude toward condoms increased significantly, irrespective of the type of person. The percentage reporting they believe their MVP completely approves condom use increased from 33% in 2000 to 37% in 2003 (see Table 1). Significant differences in perceived condom attitude were also observed by type of MVP (results not shown, χ^2^(36) = 326.9, *p *= 0.000). For instance, pooled over the three surveys 45% of youths who named friends as their MVP believe these friends approve of condom use. By contrast, only 21% of youths who named their fathers, and only 4% of those who named religious leaders, believe these persons approve of condom use.

### Bivariate relationships

Table 2 reveals a clear association between youths' perception of the MVP's approval of condom use and the frequency of condom use. Among those youths who believed that the MVP completely approved of condom use, 61% reported always using condoms with casual partners. By contrast, among youths who believed that the MPV completely disapproved of condom use, only 40% reported to always use condoms. Furthermore, the percentage of youth who never use condoms ranges from 22% among those who believe their MVP completely disapproved of condom use to 9% for those who believe their MVP completely approves it.

**Table 2:** Perceived attitude of MVP toward condom use and frequency of condom use with casual partners, pooled samples (*N *= 1587)

	Frequency of condom use with casual partners				N of cases
		
Perceived attitude of MVP toward condom use	Never	Sometimes	Often	Always	
Disapproves completely	22.4%	32.8%	5.2%	39.7%	58
Disapproves	21.9%	21.9%	14.4%	41.9%	160
Neither pro or against	12.1%	18.6%	10.5%	58.7%	247
Approves	10.4%	19.9%	12.9%	56.8%	567
Approves completely	9.4%	17.3%	11.9%	61.4%	555

Total	11.9%	19.5%	12.0%	56.6%	1,587

χ^2^(12) = 45.4, *p *= 0.000

### Structural equation models

The analysis (see Table 3) confirms the presence of homophily regarding condom attitudes. That is, respondents with more positive attitudes toward condom use are more likely to report that their MVPs support condom use. This may reflect that people tend to associate and bond preferentially with others who think alike, either as the result of selection or influence processes (actual homophily). However, it is also possible that people overestimate the extent which people they are associate with have similar attitudes (perceived homophily) [55,56]. Since homophily is expected to be strongest on divisive issues, the finding that the homophily effect decreased significantly over time suggests that there was an increased acceptance of condom use in the communities.

**Table 3:** Results of structural equation model for frequency of condom use with casual partner, MVP's perceived supports condom use, and respondent's attitude towards condom use

b (β)	MVP's perceived support of condom use			Respondent's attitude towards condom use			Frequency of condom use with casual partner		
Year	2000	2002	2003	2000	2002	2003	2000	2002	2003
*Condom attitudes and access*									
Respondent's condom attitude	9.641*** (0.719)	5.639*** (0.570)	2.083*** (0.201)				1.316***^bc^ (0.101)	1.316***^ac^ (0.132)	1.316***^ab^ (0.131)
*Social support*									
MVP's perceived support of condom use				-0.050***^b^ (-0.673)	-0.050***^a^ (-0.496)		0.078***^bc^ (0.081)	0.078***^ac^ (0.077)	0.078***^ab^ (0.081)
Discussed RH issues				0.026**^bc^ (0.087)	0.026**^ac^ (0.085)	0.026**^a^b (0.080)			0.314* (0..095)
Range of persons respondent discussed FP, STD, AIDS with							0.272** (0.158)		
Mean condom use attitude for the MVP category	0.710***^bc^ (0.088)	0.710***^ac^ (0.085)	0.710***^ab^ (0.130)						
*Socio-demographic characteristics*									
Sex (1: female)	-0.278**^bc^ (-0.066)	-0.278**^ac^ (-0.083)	-0.278**^ab^ (-0.089)	-0.029**^bc^ (-0.092)	-0.029**^ac^ (-0.085)	-0.029**^ab^ (-0.095)		0.232**^c^ (0.069)	0.232**^b^ (0.077)
Age	0.088***^bc^ (0.122)	0.088***^ac^ (0.153)	0.088***^ab^ (0.166)				0.053***^bc^ (0.075)	0.053***^ac^ (0.091)	0.053***^ab^ (0.103)
Currently in school	-0.196*^bc^ (-0.057)	-0.196*^ac^ (-0.071)	-0.196*^ab^ (-0.076)	0.027***^bc^ (0.105)	0.027**^ac^ (0.096)	0.027**^ab^ (0.107)	0.177**^bc^ (0.054)	0.177**^ac^ (0.064)	0.177**^ab^ (0.071)
Assets index				0.005*^bc^ (0.069)	0.005*^ac^ (0.062)	0.005*^ab^ (0.058)	0.037*^bc^ (0.043)	0.037*^ac^ (0.050)	0.037*^ab^ (0.047)
*Self-efficacy*									
Can convince casual partner to use condom				0.058***^bc^ (0.149)	0.058***^ac^ (0.139)	0.058***^ab^ (0.116)	1.369***^bc^ (0.268)	1.369***^ac^ (0.327)	1.369***^ab^ (0.272)
Can avoid AIDS				0.059**^bc^ (0.095)	0.059**^ac^ (0.112)	0.059**^ab^ (0.064)			
*AIDS severity*									
AIDS not a community problem	0.746* (0.155)		-0.334* (-0.084)					0.320* (0.087)	-0.467** (-0.122)
AIDS curable/not terminal				0.011*^bc^ (0.065)	0.011*^ac^ (0.063)	0.011*^ab^ (0.071)			
*AIDS risk perception*									
AIDS risk					0.016* (0.114)			0.114* (0.080)	-0.138** (-0.118)
*Sexual risk behavior*									
Number of partners, past 12 months	0.079** (0.168)						0.041***^bc^ (0.090)	0.041***^ac^ (0.117)	0.041***^ab^ (0.113)
Frequency of intercourse, past month		0.042***^c^ (0.145)	0.042***^b^ (0.132)						
Time required to obtain condom				-0.002***^b^ (-0.155)	-0.002***^a^ (-0.159)				

R^2^	7.6%	8.1%	9.8%	8.7%	11.0%	7.2%	16.3%	17.7%	18.0%

a) coefficient constrained to equal the 2000 coefficient;

b) coefficient constrained to equal the 2002 coefficient;

c) coefficient constrained to equal the 2003 coefficient

Significance: *: *p *< 0.050; **: *p *< 0.010; ***: *p *< 0.001

χ^2^(109) = 107.680, *p *= 0.518, RMSEA = 0.021, NFI = 0.968

The net effect of the respondent's perception of their MVP's support for condom use on the respondent's condom attitude is negative in 2000 and 2002, and not significant in 2003. The negative effects suggest that youths may tend to exaggerate the homophily between them and their MVPs with respect to attitudes towards condom use.

The perception that MVPs support condom use has a positive effect on youths' frequency of condom use with casual partners. The respondent's condom attitude also has a consistent positive effect on his or her frequency of condom use.

Being able to discuss reproductive health issues with others improves condom attitudes. Youths who reported discussing reproductive health issues with a large range of people are more likely than other youth to report condom use, but only in the 2000 survey. The finding that this effect was not significant in subsequent survey years may indicate that condom use is becoming more acceptable. When condoms are socially less accepted, discussing reproductive health issues with a large range of people can be interpreted as soliciting support.

Women are less likely than men to believe that their MVPs support condom use. This suggests that men and women perceive that there are different standards for men and women regarding reproductive health behaviors. While women hold less favorable attitudes toward condom use than men, they report higher rates of condom use in 2002 and 2003.

Older respondents are more likely than younger respondents to believe that their MVP supports condom use, and also report a higher frequency of condom use. Students have more favorable condom attitudes and also used condoms more frequently. Students are more likely than others to believe that their MVP disapproves of condom use.

Self-efficacy has a strong impact on condom use. Respondents who believe they can convince their partner to use condoms report a much higher frequency of condom use and have more positive attitudes towards condoms. Self-efficacy has a direct positive effect on the frequency of condom use, but also a significant indirect effect (not shown) through its effect on the respondent's condom attitude. The belief that one can avoid HIV infection also has a significant indirect effect on the frequency of condom use, again through condom attitudes.

The findings regarding the effects of perceived severity of HIV/AIDS are somewhat ambiguous. Youths who believe AIDS is very severe tend to have more negative attitudes towards condoms. In 2000, respondents who did not consider AIDS a community problem report were more likely than others to believe that their MVPs supported condom use, but by 2003 this effect has reversed. Likewise, in 2002 respondents who did not consider AIDS a major community problem reported more frequent condom use, but this effect also reversed in 2003.

As expected, youths' perceived risk of HIV/AIDS infection has a positive effect on condom use in 2002. However, the effect was negative in 2003. The number of sexual partners youths reported having in the past year has a positive effect on the frequency of condom use. The availability of and access to condoms has a positive effect on youths' condom attitudes, but they do not have a direct effect on reported condom use.

## Discussion

Our data show that condom use with casual partners increased rapidly among youth in urban Cameroon over the course of the study period. This finding is consistent with the results from the Demographic and Health Surveys [16] that reported increased condom use among married women and sexually active unmarried women. At the same time there were also significant, but less impressive, changes in attitudes towards condom use and perceived social approval of condom use.

The results show that perceived support for condom use from the respondent's most valued person has a positive effect on the frequency of condom use with casual partners. This finding is consistent with the literature on the importance of the social environment and on social support for condom use [17,33-35,37]. Information about condom use, or a favorable attitude toward it, is not sufficient to use them. The perception that condom use is approved of by people whose opinion one values is a crucial link in the process of adopting condom use.

Although the perception that MVPs support condom use only had a small effect, it is noteworthy that youths rarely named a peer as someone whose opinion mattered much to them. The overwhelming majority named their parents or other relatives as MVPs. This emphasizes the importance of vertical over horizontal relations, and of traditional authority relations. Peers may be important for knowledge and such, but in the end it is still the approval of parents and relatives that matters. AIDS prevention programs, therefore, should pay more attention to these traditional authority structures as any widespread behavior change requires support of both important adults and peers. Recognizing the importance of these authority structures, the *100% Jeune *program increased their focus on parent-youth communication. Rather than focusing on specific target groups, such as youth and other high risk groups, AIDS programs should be more encompassing and include the entire community. Whenever possible, traditional authority systems should be mobilized to promote and support behavior change. The finding that the negative effect of the MVP's opinion on the respondents' condom attitudes disappeared by the end of the study period suggests that this approach may be having success. Improved communication, especially with parents, creates an environment that facilitates the adoption of reproductive health behaviors, in which youths feel support for their decisions.

## Conclusion

Because people prefer to associate with others who are similar, homophily is a major organizing principle in human relations. Hence, many public health programs assume that peers are the relevant reference group for adolescents. However, the homophily principle applies only to voluntary relationships when there are few structural constraints on one's relationships. Relationships with family and the community are ascribed, not achieved. While peers may influence one's beliefs and knowledge, it is the non-voluntary relationships with family and community members that exert social control over the behaviors of adolescents. This, however, does not suggest that peers are irrelevant for the adoption of condoms or that peer education programs should be abandoned. Peers remain a very important reference group for adolescents. The study does suggest, however, that although peers may be a source of social approval and support, their support is insufficient for the adoption of condom use. Other actors, primarily parents and other family members, play a crucial role in this respect. This suggests a two-pronged approach: a traditional program oriented towards adolescents, and a second program that targets the community with special attention to opinion leaders in the community. The role of these opinion leaders is twofold: disseminating information about safe sex behaviors, and providing moral approval for such behaviors. As there is no reason to belief that these findings are unique to these Cameroonian youth [see e.g. reference [36]] such two-pronged approach may be applicable in many more communities worldwide.

This study also has its limitations, several of which are related to the question about the MVP. Respondents were only allowed to name a single MVP, while social approval is an environmental process involving multiple persons. The results therefore confirm the importance of parents and family, but they do not necessarily prove that peers are unimportant. It is quite possible that if more choices were allowed, peers would have been mentioned as second or third choice. Second, the question asked respondents to identify someone whose opinion they valued; it did not specifically ask whose opinion they valued with respect to reproductive health issues or condom use. Had they been asked whose opinion they values for reproductive health issues, they may have named a different person. Nevertheless, we did observe that the perception that the person named as MVP approved condom use had a significant effect on condom use. If our measure of perceived social approval was invalid, either because it either did not include all relevant persons or was too general, then one would expect to find no effect of this variable on the outcome. Although social approval is a community process, not everyone is equally important. Social approval is linked to the stratification within the community and the status of individuals. Because youths tend to have lower status they may be less important for social approval. Furthermore, because of its link to the stratification system, the social approval system also tends to be more general rather than issue-specific.

Although the use of structural equation models allowed us to both include a non-recursive element in the model and to compare regression coefficients across the three survey waves, it only estimates linear regression equations. For the ordinal variables in the model we therefore had to use polychoric correlations. As the polychoric correlation assumes an underlying normal distribution of the variable, the extent to which this assumption does not hold might affect the estimated coefficients.

Further, this study uses a series of cross-sectional surveys rather than longitudinal data which means we cannot assess individual behavioral change or measure the role of social approval in the change process. Because our study only includes youths who reported having casual partners in the past 12 months, our findings may not apply to those who only have regular partners. The role of MVPs and perceived social approval may be different in these groups as decision making takes place in different contexts.

Although there is little data on the frequency of condom use in Cameroon, the DHS data indicate that condom use has increased substantially since the mid-nineties. Our study falls in the middle of this period and provides additional evidence for such a change. Not only did condom use become much more frequent from 2000 to 2003, but attitudes shifted to being more favorable towards condoms as well, and MVPs were reported to be more supportive of condom use. The changes in attitudes, however, are fairly small compared to changes in behavior. When support for condom use becomes widespread, one can, paradoxically, expect social support to become a less important factor regarding condom use. In this study, however, no such shift in the effect of social support was yet observed. This implies that for the time being programs should keep focusing on building widespread support for condom use in the general population, and not simply focus on groups-at-risk.

## Competing interests

The authors declare that they have no competing interests.

## Authors' contributions

RVR designed the study, performed the statistical analysis and drafted the manuscript. DM co-designed the study, directed the field work and helped to draft the manuscript. Both authors read and approved the final manuscript.

## Appendix 1

**Items included in condom attitude scale**.

#      Item

1   Seriez-vous gêné d'acheter des condoms dans une boutique à côté de votre domicile? (Would you be embarrassed to buy condoms in a shop near your residence?)

2   Seriez-vous gêné de discuter de l'utilisation des condoms avec vos amis? (Would you be embarrassed to discuss the use of condoms with your friends?)

3   Croyez-vous que les femmes qui ne sont pas mariées devraient toujours être en mesure d'acheter des condoms? (Do you believe that non-married women should always be able to buy condoms?)

4   Les condoms sont-ils efficaces dans la prévention des maladies sexuellement transmissibles? (Are condoms effective for the prevention of sexually transmitted diseases?)

5   Les condoms sont-ils efficaces pour prévenir le VIH/SIDA? (Are condoms effective for the prevention of HIV/AIDS?)

6   Pensez-vous que les condoms se rompent facilement? (Do you think condoms rip easily?)

7   Pensez-vous qu'il est difficile d'utiliser le condom avec une personne avec qui vous ne l'avez jamais utilisé avant? (Do you think it is difficult to use a condom with someone with whom you never used one before?)

8   Pensez-vous qu'il est facile d'introduire les condoms en disant qu'il est pour la prévention des grossesses non désires? (Do you think it is easy to introduce condoms by stating it is for the prevention of unwanted pregnancies?)

9   Pensez-vous que les condoms sont difficiles à obtenir? (Do you think condoms are difficult to obtain?)

10   Une femme peut-elle demander qu'un homme utilise le condom pour une raison quelconque? (Can a woman ask a man to use a condom for whatever reason?)

11   Pensez-vous que l'utilisation du condom diminue le plaisir sexuel pour l'homme? (Do you think that using a condom reduces the sexual pleasure of the man?)

12   Pensez-vous que l'utilisation du condom diminue le plaisir sexuel pour la femme? (Do you think that using a condom reduces the sexual pleasure of the woman?)

13   Pensez-vous que le condom peut resté dans le vagin de la femme au cours de son utilisation? (Do you think a condom may remain in a woman's vagina after use?)

14   Pensez-vous que le condom peut irriter ou blesser le vagin de la femme au cours de son utilisation? (Do you think a condom may irritate or hurt a woman's vagina during use?)

15   Pensez-vous qu'on ne devrait pas faire confiance à un partenaire qui suggère le port du condom? (Do you think one cannot trust a partner who suggest the use of a condom?)

16   Une femme peut-elle se protéger contre une MST si son mari en est déjà atteint? (Can a woman protect herself against a STI when her husband is already infected?)

Each item was scored 0: no, 1: yes and 0.5: do not know/no opinion. All items were (re-)scored such that a higher total score reflect a more positive attitude towards condom use.

## Pre-publication history

The pre-publication history for this paper can be accessed here:

http://www.biomedcentral.com/1471-2458/11/632/prepub
